# Predictors of Non-Sentinel Nodal Involvement in Breast Cancer Patients with Positive Sentinel Nodes Undergoing Upfront Surgery

**DOI:** 10.12669/pjms.41.6.11876

**Published:** 2025-06

**Authors:** Muhammad Usman Tariq, Sana Zeeshan, Aiman Arif, Lubna Vohra, Romana Idrees

**Affiliations:** 1 Muhammad Usman Tariq Section of Histopathology, Department of Pathology and Laboratory Medicine, Aga Khan University, Karachi, 74800, Pakistan; 2 Sana Zeeshan Section of Breast Surgery, Department of Surgery, Aga Khan University, Karachi, 74800, Pakistan; 3 Aiman Arif Department of Surgery, Aga Khan University, Karachi, 74800, Pakistan; 4 Lubna Vohra Section of Breast Surgery, Department of Surgery, Aga Khan University, Karachi, 74800, Pakistan; 5 Romana Idrees Section of Histopathology, Department of Pathology and Laboratory Medicine, Aga Khan University, Karachi, 74800, Pakistan

**Keywords:** Breast cancer, Sentinel lymph node biopsy, Non-sentinel lymph node, Axillary dissection, Lymph node metastasis

## Abstract

**Background & Objective:**

Sentinel lymph node (SLN) biopsy is performed to stage the axilla in clinically node negative breast cancer (BC) and is converted to axillary lymph node dissection (ALND) if found to be positive for tumor metastasis. A significant proportion of positive SLN cases do not reveal any positive non-SLN (NSLN) emphasizing the need to evaluate other clinicopathological factors which could better predict NSLN involvement and avoid unnecessary ALND in a select group of patients. We aimed to find the correlation of clinicopathological features of primary BC, positive SLN and NSLN after ALND in order to identify factors that may help in predicting involvement of NSLN.

**Methods:**

One hundred thirty BC cases with positive SLN who underwent ALND at Aga Khan University (2010-2022) were retrospectively reviewed for clinicopathological features to assess their association with status of NSLN involvement.

**Results:**

On univariate analysis, positive SLN to total SLN ratio, axilla scoring system (ASS) score and area of the largest metastatic tumor focus appeared as significant factors. However, area of the largest metastatic tumor focus was the sole predictor to NSLN involvement on multivariate analysis (odds ratio: 1.01). Chi-square test was used to determine that NSLN positivity was significantly associated with a positive SLN to total SLN ratio of >0.6, area of largest metastatic focus of >50mm^2^ and ASS score of >5.5.

**Conclusion:**

This study highlights the need to consider other pathological factors (in addition to macrometastasis) before making the decision of ALND after positive SLN.

## INTRODUCTION

Ever since the acceptance of sentinel lymph-node biopsy (SLNB) as standard of care in clinically node negative axilla in early breast cancer (BC), its indications have now expanded to larger tumors, ductal carcinoma in-situ (DCIS) and post neoadjuvant settings.^1^-^3^ Several institutions worldwide, particularly with a multidisciplinary team and dedicated breast pathologists, have demonstrated SLNB to be highly accurate in predicting axillary tumor burden in patients with BC.^4^ Axillary lymph-node dissection (ALND) has previously been the standard of care for axillary nodal involvement after SLNB. However, according to literature, the possibility of involvement of non-sentinel lymph nodes (NSLN) on complete ALND after positive SLNB varies greatly (27-68%), with some reports stating involvement of only SLN in up to 70% cases.^4^-^6^ Recent trials such as AMAROS, have proven equal efficacy of axillary radiotherapy as compared to ALND after positive SLNB in cases of early breast cancer, T1-T2 disease and no palpable axillary lymphadenopathy. This has also been incorporated in the latest National Comprehensive Cancer Network (NCCN) guidelines.^7^

Involvement of NSLN with positive SLN may depend upon various factors, such as size and biology of primary tumor, lymphovascular invasion (LVI), number and ratio of positive SLN, size and number of metastatic tumor deposits in positive SLN and extranodal/capsular extension, amongst few others.^4^,^6^,^8^ Of these, the number of positive SLN, the ratio between positive SLN and total number of SLN retrieved, extranodal extension and LVI were significant predictors for NSLN involvement in most studies.^4^,^6^-^11^ Based on these findings, few nomograms and axillary scoring systems have also been developed claiming to assist surgeons in predicting the NSLN status in BC patients with SLN involvement.^8^,^11^

The lack of involvement of NSLN in up to 70% of cases raises the question of performing unnecessary axillary dissection in a group of patients who would have otherwise benefitted without additional surgery and avoided its adverse effects. At our institution, the current practice continues to be performing ALND after positive SLNB in all patients regardless of their stage and the type of breast surgery. This study was conducted to identify clinicopathological features of primary tumor and positive SLN which would be helpful in predicting NSLN involvement and safely avoiding ALND in patients with less likelihood of NSLN involvement.

### Primary objective:

To study the effect(s) of number, location, size of metastatic tumor and extranodal extension of positive axillary SLN in predicting positivity of NSLN.

### Secondary objective:

To study the relation of clinicopathological features such as patient’s age, tumour type, tumour grade, primary tumor size, lymphovascular invasion, and axilla scoring system with positivity of NSLN.

## METHODS

This retrospective cross-sectional study was conducted at the Section of Breast Surgery of Department of Surgery and Histopathology Section of Pathology and Laboratory Medicine Department of Aga Khan University Hospital, Karachi.

### Ethical Approval:

Since this study didn’t involve patients directly, institutional ethics review committee granted exemption from approval (Study ID: 2020-4886-10600).

### Inclusion criteria:

- Patients who underwent upfront surgery, either mastectomy or breast conservation surgery (BCS), along with ALND after histological confirmation of positive SLN during January 2010 – April 2022.
- Patients who had impalpable nodes on clinical examination of the axilla and appeared benign on ultrasound and those who had suspicious nodes on ultrasound but were proven benign after biopsy.

### Exclusion criteria:

- Patients who received neoadjuvant treatment and SLN with micrometastasis or isolated tumor cells.

Based on the aforementioned eligibility criteria, we included 130 BC cases in our study. The frozen section process involved slicing of SLN into 2-3 mm sections before submitting entirely. After freezing at -24°C in cryostat, 3 sections of 4-micron thickness were taken at intervals of 500 to 1000-microns. The sections were stained with hematoxylin-and-eosin (H&E) stain and immunohistochemistry was not performed at the time of frozen section. Size of the largest metastatic focus is only single (largest) dimension of the largest metastatic tumor focus. While the area of the largest metastatic tumor focus is the product of two dimensions of the area occupied by the largest metastatic tumor focus. This area provides better idea of burden of metastatic disease. Location of the metastatic tumor focus was recorded as “subcapsular”, “intraparenchymal” or “both”.

The microscopic glass slides of SLN were independently reviewed for 115 cases by two histopathologists for histological features described in primary objective. The slides of NSLN were reviewed for presence of metastatic tumor. The findings were entered on a predesigned proforma and then transferred to SPSS, Version 21. Data regarding patient’s age, gender, tumor type, tumor grade, primary tumor size and LVI were recorded from patient’s charts and institution’s breast cancer database. The distinction between luminal A and luminal B was based on Ki-67 index. Tumors with Ki-67 index < 20% were labeled as luminal A, while tumors with Ki-67 index ≥ 20% were labelled as luminal B. Each case was scored according to Axilla scoring system (ASS) as suggested by Barranger et al.^8^

ASS is a scoring nomogram devised by Barranger et al with the purpose of assigning a score to patients that can predict the presence of positive NSLN in patients with positive SLN upon frozen section analysis.^8^ Patients with a score of ≤ 3.5 had a 97.3% chance of having negative NSLN based on factors such as presence of macrometastases, histological tumor size and ratio of positive to total SLNs. Mean ± standard devation or median and range (as appropriate) were calculated for quantitative variables including age, primary tumor size, metastatic tumor size, area covered by metastatic tumor, number of metastatic tumor foci, total number of SLN and number of positive SLN. Ratio of number of positive SLN to total number of SLN was calculated. Frequency and percentages were reported for number of patients with positive NSLN, location of metastatic tumor focus, extranodal extension, tumor type, tumor grade, pathologic T size, LVI and status according to axilla scoring system. Univariate analysis was performed to assess the relationship of all clinicopathological and histological features with status of NSLN involvement by applying logistic regression and crude odds ratio was calculated. Multivariate logisitic regression was carried out only for variables that showed significant association on univariate analysis and adjusted odds ratio was calculated for them. A *p*-value of <0.05 was considered significant throughout the study. Chi-square test was applied to significant variables at different cut-offs to determine the cut-off value that demonstrated significance with NSLN positivity.

## RESULTS

A total of 130 cases were included in the study. The clinicopathological features of the patients are summarized in Table-I. Mean age of the patients was 54.9 ± 12.6 years and all patients were female. Mean tumor size was 3.3 ± 1.7cm. Invasive carcinoma of no special type [previously known as invasive ductal carcinoma (IDC)] was the most common histologic type of breast carcinoma (86.2%), followed by invasive lobular carcinoma (11.5%). Almost two third tumors were grade II. Majority of the tumors qualified as T2 (70%) and N1a (81.5%). LVI was seen in 32.3% cases. Luminal A was the most common molecular subtype (64.6%) followed by Luminal B (19.2%). Majority (83.1%) of the patients underwent mastectomy.

**Table-I:** Summary of clinicopathological features of breast cancer patients.

Clinicopathological features (n=130)	Expression (%)
***Patient’s age (years):***	
• Mean (SD)	54.9 (12.6)
• Range	26 - 90
***Tumor size (cm):***	
• Mean (SD)	3.3 (1.7)
• Range	0.5 - 10.5
***Histologic type:***	
• Invasive carcinoma of no special type / IDC	113 (87%)
• Invasive lobular carcinoma	15 (11.5%)
• Metaplastic carcinoma	2 (1.5%)
***Histologic grade:***	
• Grade I	8 (6.2%)
• Grade II	83 (63.8%)
• Grade III	39 (30%)
***cT stage:***	
• T1	21 (16.2%)
• T2	91 (70%)
• T3	17 (13.1%)
• T4	1 (0.8%)
***Lymphovascular invasion***	
• Present	42 (32.3%)
• Absent	88 (67.7%)
***Molecular subtype:***	
• Luminal A	84 (64.6%)
• Luminal B	25 (19.2%)
• Her2*neu* enriched	5 (3.8%)
• Triple negative	7 (5.4%)
• Not known	9 (6.9%)
***Type of Surgery:***	
• Mastectomy	108 (83.1%)
• Breast Conservation Surgery	22 (16.9%)

The pathological features of the SLN and NSLN are summarized in Table-II. The number of SLN received in each case ranged from 1- 11 and median was Three. The number of positive lymph nodes ranged from 1-8 and median was 1. Ratio of positive SLN to total SLN ranged from 0.13-1. Additional histologic features were reviewed for 115 cases. Size and area of the largest metastatic focus ranged from 3-25 mm and 1-330 mm^2^ respectively. Number of metastatic foci in the largest lymph node ranged from 1-10 and the median was One. Majority of the SLN showed metastatic tumor foci in subcapsular and intraparenchymal location (58.2%). Extranodal extension was seen in 47.7% cases. The number of NSLN received in each case ranged from 1-47 and median was 15.5. NSLN involvement was seen in 36.9% cases. The number of positive NSLN in each case ranged from 1-26 and median was Two. We applied the axilla scoring system (ASS) as proposed by Barranger et al.^8^ and scores of our cases ranged from 2-7 with median of 6. While ASS included separate points for presence of macrometastases, micrometastasis and isolated tumor cells within SLN, all our patients had macrometastases as per our inclusion criteria, therefore, all patients were given the two points for presence of macrometastases.

**Table-II:** Summary of pathological features of sentinel and non-sentinel lymph nodes. (n=130)

Pathological featuresn=130	Expression
***Total number of SLN in each case***	
• Median (Range)	3 (1-11)
***Number of positive SLN in each case***	
• Median (Range)	1 (1-8)
***Ratio of positive SLN to total number of SLN***	
• Median (Range)	0.5 (0.13-1)
***Size of the largest metastatic focus (mm)***	
• Mean (SD; Range)	7.4 (4.5; 3-25)
Area of the largest metastatic focus (mm^2^) (n=115)*	
• Mean (SD; Range)	52.9 (64.2; 3-330)
Number of metastatic foci in the largest positive SLN (n=115)*	
• Median (Range)	1 (1-10)
Location of metastatic foci in the largest positive SLN (n=115)*	
• Subcapsular only	27 (23.5%)
• Intraparenchymal only	21 (18.3%)
• Subcapsular and Intraparenchymal	67 (58.2%)
***Extranodal extension:***	
• Present	62 (47.7%) 68 (52.3%)
• Absent	
***Total number of NSLN in each case***	
• Median (Range)	15.5 (1-47)
***NSLN involvement***	
• Present	48 (36.9%)
• Absent	82 (63.1%)
***Number of involved NSLN in positive cases***	
• Median (Range)	2 (1-26)
Axillary scoring system	
• Median (Range)	6 (2-7)

* Slides of 115 cases were available for reviewing the additional histological features including area of the largest metastatic focus, number of metastatic foci and location of metastatic focus in the largest sentinel lymph node.

Univariate analysis was done for all variables and showed significant association of NSLN positivity with ratio of positive SLN to total number of SLN (OR: 3.54), area of the largest metastatic focus (OR:1.01) and the score as per ASS (OR: 1.45) at *p-*value <0.05. The ASS was adapted from the Tenon model that incorporated pathologic tumor size, ratio of positive SLN to total number of SLN and the presence of macrometastasis in SLN (which as mentioned previously was present in all our patients).^8^ Multivariate analysis was done for these three variables with significance on univariate analysis and showed significant association between area of largest metastatic focus and NSLN positivity when adjusted for ratio and ASS score. (OR: 1.01; *p-*value = 0.023). This means that there were a 1% increase in the likelihood of NSLN positivity for every mm^2^ increase in area. The rest of the factors were not significant on univariate analysis; hence, they were not included in the multivariate analysis (Table-III). Chi-square test was used to assess the relationship between NSLN positivity and significant variables. Different cut-off values were used for analysis and significant association was seen with ratio of positive to total SLN > 0.60, area of the largest metastatic focus > 50mm2, and axilla scoring system ≥ 5.5. (Table-IV).

**Table-III:** Univariate and Multivariate Analysis for significant features.

Clinicopathological features	Crude odds ratio (95% CI)	P-value	Adjusted odds ratio (95% CI)	P-value
Ratio of positive SLN: Total SLN	3.54 (1.06-11.79)	.04	1.98 (0.24-16.38)	.53
Area of largest metastatic focus (mm2) (n=115)*:	1.01 (1.00-1.02)	.011	1.01 (1.00-1.02)	.023
Axillary Scoring System	1.45 (1.01-2.07)	.043	1.10 (0.61-1.98)	.76
Tumor Size (cm)	0.97 (0.78-1.20)	.79	-	-
Lymphovascular Invasion	1.95 (0.92-4.14)	.083	-	-
Size of the largest metastatic focus (mm):	1.07 (0.99-1.16)	.100	-	-
***Histologic Grade***				
Grade I (ref)	-	.261	-	-
Grade II	0.36 (0.08-1.60)	.179		
Grade III	0.27 (0.06-1.30)	.102		

**Table-IV:** Chi-square tests for significant variables at their cut-offs.

Clinicopathological features	NSLN positive	NSLN negative	P-value
***Ratio of positive to total number of SLN:***			
• ≤ 0.60	22 (29.3%)	53 (70.7%)	.036
• > 0.61	26 (47.3%)	29 (52.7%)	
Area of the largest metastatic focus (n=115)*:			
• 3-50 mm^2^	22 (27.8%)	57 (72.2%)	.021
• > 50 mm^2^	18 (50%)	18 (50%)	
***Axilla scoring system:***			
• < 5.5	14 (25.5%)	41 (74.5%)	.020
• ≥ 5.5	34 (45.3%)	41 (54.7%)	

## DISCUSSION

SLN biopsy is a fundamental component for axillary staging in early BC with clinically negative axilla. Recent literature has focused more on finding evidence to minimize ALND as much as possible to eliminate the potential side effects and morbidity associated with it.^12^ The ACOSOG Z0011 and multiple subsequent trials have reported that overall survival in patients with SLN metastases undergoing SLN biopsy alone is not inferior to those undergoing SLN biopsy and ALND in patients with adjuvant systemic and radiation therapy.^5^,^13^,^14^ Moreover, the Z0011 trial also reported a higher number of adverse surgical effects in patients undergoing SLN biopsy + ALND (70%) as compared to patients undergoing SLN biopsy alone (25%). These adverse effects included wound infections, seroma, paresthesias and lymphedema.^15^ With up to 70% of patients having negative NSLN on ALND after positive SLN biopsy, and comparable overall survival in both groups, determining patients in which ALND can be avoided with minimal risk of recurrence can greatly impact the post-operative outcomes of those patients.^15^

Our study demonstrated a NSLN positivity rate of 36.9% with a median of two positive NSLN in NSLN positive patients. This shows that 63.1% patients with positive SLN biopsy did not have further positive axillary lymph nodes. Our results are comparable to the study done by Maher et al who reported 63.4% patients with no other positive nodes beyond SLN after completion ALND.^5^ Another study done on 443 SLN positive patients also detected 62.3% patients without metastases in NSLN.^16^ This reinforces the need to devise methods to eliminate unnecessary extensive axillary surgery. A lot of efforts have been made to de-escalate axillary surgery especially with trials like AMAROS and Z0011 that have proved the non-inferiority of omitting ALND in favour of axillary radiation in early breast cancer patients with positive SLN. These trials have informed guidelines that have reduced the number of patients undergoing unnecessary ALND. However, the surgeons in our institution still follow previous practice of performing ALND after a postive SLNB. Another possible explanation for this practice is the higher frequency of mastectomy as compared to conservation despite 86.2% patients having ≤ T2 disease and not requiring radiation.^7^,^17^

This study demonstrates a significant relationship between positive NSLN and the ratio of positive SLN to total SLN, as indicated by the results of our univariate analysis. The proportion of SLN involvement was also a significant predictor in the study reported by Barranger et al.^8^ Our study was able to show significant association of NSLN positivity with a ratio of positive SLN to total SLN of > 0.61. However, it failed to show a significant association between NSLN status and the ratio in the multivariate analysis. Wang et al. also reported that a SLN positivity rate greater than 0.33 was associated with a greater number of NSLN patients with a sensitivity of 71.4% and specificity of 65%.^18^ Another study reported significance at a cut-off of 0.5 with an adjusted odds ratio of 2.86 (P = 0.024).^19^

Although size of the largest metastatic focus did not show a significant association in our study, area of tumor deposit was the only predictor of NSLN status with significant results in both univariate and multivariate analysis. It also showed significant association at cut-off >50mm^2^. While size of metastatic foci has been implicated in a number of studies,^8^,^17^,^20^ area has not been studied in literature well and therefore, more research on this factor may lead to a better understanding of its role in predicting NSLN status. We think that multiple tumor foci better represent metastatic disease burden and therefore it can better predict non-SLN involvement.

We used the ASS designed by Barranger et al. and applied it to our dataset.^8^ A significant change in the positivity of NSLN was seen with an increase in the axillary score in the univariate analysis (OR: 1.45, P = 0.043). Since only the presence of macrometastasis in SLN warrants ALND and is the standard of care, micrometastasis and isolated tumor cells in SLN were not present in our study, hence, every patient was given two points for presence of macrometastases. We were also able to demonstrate that NSLN positivity was significant at ASS ≥5.5. This shows that while primary tumor size in isolation was unable to show a significant correlation with NSLN status, it did give significant results when combined with the ratio of positive SLN in our modified axillary scoring system.

Other nomograms developed in literature have also incorporated similar predictors for NSLN positivity. Murata et al. used clinical tumor size, axillary ultrasound findings, histologic type, size of SLN metastasis, ratio of the number of positive SLN to the total number of SLN removed, and number of positive SLNs, and reported an area under curve of 90% for their validation cohort with a 96.9% negative predictive value at cut-off value of 4.^19^

Our study did not show a significant association between NSLN status and histologic grade and type of tumor, primary tumor size, molecular subtype, size of the largest metastatic focus in the SLN, LVI, extranodal extension or location of metastatic focus in the largest positive SLN. These results may be due to a low sample size of our study. These factors have been reported to be significant predictors for axillary lymph node status in other studies.^21^,^22^ Xiong et al. reported that Luminal B subtype was significantly more likely to lead to axillary lymph node metastases.^22^ Histologic grade has also shown significant relationship with axillary node status with grade III having the strongest association.^21^,^22^

Primary tumor stage has been shown to be one of the strongest predictors of NSLN status in multiple studies with a significant increase in NSLN involvement when tumor stage changed from T1b to T2.^8^,^23^ Other studies have used clinical or pathological tumor size to predict NSLN status.^19^,^20^,^22^ Following the ACOSOG Z0011 trial, the NCCN recommended no further axillary surgery in patients with T1-T2 tumors, less than three positive SLNs, who undergo lumpectomy without preoperative systemic therapy and mandatory radiation therapy postoperatively.^18^,^20^ Other factors that have been reported to show significant association with axillary lymph node metastases include LVI and lymph node mass descriptors such as cortical thickness and shape.^22^,^24^

ALND is not only associated with increased morbidity pertaining to reduced arm mobility, lymphedema and increased susceptibility to infections, but it is also associated with increased health care costs.^25^ This study is the first of its kind from Pakistan and may help lay the groundwork for a potential local randomized control trial by developing further nomograms that could lead to avoiding unnecessary ALND after positive SLN biopsy with negative NSLN. Moreover, our study reflects upon the current practice of surgeons in terms of performing ALND indicating significant number of cases where ALND could have been avoided mandating change of practice. Our study also highlights the importance of further exploring the potential role of measuring the area of the largest metastatic tumor focus in SLN which can help in predicting NSLN status as it is an underexplored factor in published literature.

### Limitations

The limitations of this study are that it was a single-center retrospective study with a relatively smaller sample size and therefore, more data may be required for it to be generalized to the entire population.

## CONCLUSION

****In a significant number of BC patients, SLN are the only involved lymph nodes and an extensive ALND yields only negative NSLN which points to the fact that the current threshold/cut-off value of 2mm is not efficient enough to predict NSLN involvement and raises the need to evaluate other pathological factors. We identified area of the largest metastatic tumor focus to be the only factor predicting NSLN positivity on multivariate analysis. Further studies are required on larger cohorts to validate and generalize this finding.

### Ethical Considerations:

This study did not involve patients directly, no additional tests were applied and the entire data was collected from medical records and biopsy material already submitted for diagnostic purpose, hence, exemption was given from the Ethical Review Committee (ERC) of The Aga Khan University Hospital, Karachi. The data was coded and is stored in a password protected folder in PI’s office desktop for seven years after completion of study.
